# Isoform-Specific NO Synthesis by *Arabidopsis thaliana* Nitrate Reductase

**DOI:** 10.3390/plants8030067

**Published:** 2019-03-16

**Authors:** Marie Agatha Mohn, Besarta Thaqi, Katrin Fischer-Schrader

**Affiliations:** Institute of Biochemistry, Department of Chemistry, Zülpicher Str. 47, University of Cologne, 50674 Cologne, Germany; mmohn@smail.uni-koeln.de (M.A.M.); bthaqi@smail.uni-koeln.de (B.T.)

**Keywords:** nitrate reductase, NIA1, NIA2, nitric oxide, nitrite, nitrate, methyl viologen, benzyl viologen, NO analyzer, molybdenum cofactor, *Arabidopsis thaliana*

## Abstract

Nitrate reductase (NR) is important for higher land plants, as it catalyzes the rate-limiting step in the nitrate assimilation pathway, the two-electron reduction of nitrate to nitrite. Furthermore, it is considered to be a major enzymatic source of the important signaling molecule nitric oxide (NO), that is produced in a one-electron reduction of nitrite. Like many other plants, the model plant *Arabidopsis thaliana* expresses two isoforms of NR (NIA1 and NIA2). Up to now, only NIA2 has been the focus of detailed biochemical studies, while NIA1 awaits biochemical characterization. In this study, we have expressed and purified functional fragments of NIA1 and subjected them to various biochemical assays for comparison with the corresponding NIA2-fragments. We analyzed the kinetic parameters in multiple steady-state assays using nitrate or nitrite as substrate and measured either substrate consumption (nitrate or nitrite) or product formation (NO). Our results show that NIA1 is the more efficient nitrite reductase while NIA2 exhibits higher nitrate reductase activity, which supports the hypothesis that the isoforms have special functions in the plant. Furthermore, we successfully restored the physiological electron transfer pathway of NR using reduced nicotinamide adenine dinucleotide (NADH) and nitrate or nitrite as substrates by mixing the N-and C-terminal fragments of NR, thus, opening up new possibilities to study NR activity, regulation and structure.

## 1. Introduction

In higher land plants, nitrate is the preferred nutrient for the nitrogen (N) assimilation pathway [1,2]. Nitrate reductase (NR, EC 1.7.1.1), which catalyzes the first intracellular and rate-limiting step in nitrate assimilation, is a homodimer of two approximately 100 kDa polypeptide chains, each of which binds three cofactors in individually folded domains. The enzyme functions as an internal electron transport chain [1]. The C-terminal domain carrying a flavine adenine dinucleotide (FAD) cofactor accepts two electrons from NADH or the phosphorylated form NADPH and passes them sequentially to the middle domain containing a *b*_5_-type cytochrome heme. From here, the electrons are shuttled to the molybdenum cofactor (Moco)-containing catalytic site in the N-terminal domain, and it is here that substrate reduction takes place. Three non-conserved flexible regions are found in NR: an N-terminal peptide preceding the Moco-domain, and two linkers connecting the central heme-domain to the Moco-domain (hinge 1) and to the FAD-containing domain (hinge 2). Hinge 1 in NRs of higher plants has been demonstrated to be crucial for reversible inhibition of NR at the protein level [3,4]. A highly-conserved phosphoserine residue in hinge 1, as well as a motif rich in acidic residues in the N-terminal peptide, are the targets for binding of one of several 14-3-3 protein isoforms, which leads to inhibition by steric hindrance of the internal electron transfer between the heme and Moco [5,6]. 

In addition to its main anabolic function, NR has also been proposed to act as nitrite reductase in plants resulting in the formation of nitric oxide (NO) [7,8,9]. This universal signaling molecule is involved in various physiological processes in plants, such as development or stress responses (reviewed in [10,11,12]). Several NO sources are known in plants, among them the non-enzymatic, pH-dependent NO formation in the apoplast [13], an arginine-dependent oxidative reaction mechanism observed in peroxisomes and chloroplasts similar to the nitric oxide synthase activity of mammals [14,15], and NO synthesis based on heme-proteins or the mitochondrial respiratory electron transport chain [16]. All other members of the Moco-enzyme family, to which NR belongs, namely sulfite oxidase (SO), xanthine oxidoreductase/dehydrogenase (XOR), aldehyde oxidase (AO), and the amidoxime-reducing component (ARC) were shown to be capable of NO synthesis besides their respective name-giving functions (reviewed in [17]). 

Several studies using plants, plant cells, or nitrate reductase purified from native tissue have in part quantified the NO formation by NR and suggested that it represents the major enzymatic source of NO [18,19,20,21]. Recent studies with the eukaryotic algae *Chlamydomonas reinhardtii* demonstrated that NR is also able to transfer electrons from its C-terminal FAD cofactor directly to other proteins, such as truncated hemoglobins (THB) or ARC [22,23]. While *Chlamydomonas* THB1 has an NO dioxygenase activity that consumes NO, ARC can act as an NO synthase. This finding, together with the observation that both NR and ARC are co-regulated on the transcriptional level, and that the NO synthesizing function of ARC is not inhibited by high nitrate concentrations (in contrast to plant NR, for which a K_i_^nitrate^ of 50 µM for the nitrite reductase activity was observed [24]), allowed the authors to propose that this physiologically relevant NO synthase in *Chlamydomonas* might be made up of two proteins, NR and ARC, forming a catalytic complex. Consequently, they suggested renaming ARC to NO-forming nitrite reductase (NOFNiR) [22]. Considering that NR is also involved in the removal of NO, these findings underline the complex role of NR in NO homeostasis (reviewed in [25]). 

Interestingly, the function of NR in plants becomes even more complicated by the fact that many plants including *Nicotiana tabacum, Hordeum vulgare*, *Zea mays*, *Brassica napus*, *Glycine max*, *Oryza sativa* or *Arabidopsis thaliana*, possess two or more isoforms of NR, which might take over distinct functions. For some isoforms, it is known that they differ in their preference for the co-substrate NADH or NADPH [26,27], and in some plant species, the existence of both constitutively expressed and inducible NR isoforms was reported [28]. 

Focusing on the model plant *Arabidopsis thaliana*, the transcription of the two isoforms *NIA1* and *NIA2* is similar following the induction by nitrate, while several other factors including light or the cytokinin benzyladenine produce specific expression patterns for each isoform [29,30,31].

In plant extracts of *Arabidopsis thaliana*, it is impossible to differentiate between the proteins NIA1 and NIA2 that share 78% sequence identity, because only antibodies recognizing both isoforms are commercially available. Therefore, studies on the differences between NIA1 and NIA2 have mainly relied on mutant plants, in which one and/or the other *NIA* gene has been knocked out. Based on functional analyses of these mutant plants, some differences between NIA1 and NIA2 activity at the whole-plant level have been identified. For example, it was found that *nia2* knockout plants have only 10 to 20% residual nitrate reduction activity [32,33], or while ABA-induced NO synthesis to mediate guard cell closure was attributed to NIA1 [34], others report that both NR isoforms contributed to salicylic acid-induced NO production, mediating stomatal closure [35]. 

Information on the biochemical level about distinct functions of the NR isoforms is lacking to date. Therefore, we have established in vitro systems to analyze both the nitrate and nitrite reduction activities of plant NR. We produced functional proteins of the two NR isoforms from *A. thaliana* and subjected them to steady-state enzymatic studies to characterize their functional properties. We found that both isoforms are able to use either nitrate or nitrite as a substrate, with NIA2 having a clear preference for nitrate reductase activity, while NIA1 is the more efficient nitrite reductase, and the nitrite reducing activities of both were inhibited at low concentrations of nitrate.

## 2. Results

### 2.1. Nitrate Reduction Activity

NR is modularly folded and individual domains retain a partial activity of the full-length protein [36,37,38]. We have shown in the past that the N-terminal fragment of *Arabidopsis thaliana* NIA2 comprising the Moco- and heme-domains connected by hinge 1 (residues 1–625, NIA2-Mo-heme) exhibits similar nitrate reduction activity and 14-3-3 protein-mediated inhibition properties to the full-length NIA2 when the artificial electron donor reduced methyl viologen (MV) is supplied for nitrate reduction [5,6]. Therefore, we produced the corresponding N-terminal fragment of NIA1 (residues 1–627, NIA1-Mo-heme) to compare it to the kinetic properties of purified NIA2-Mo-heme. 

**Figure 1:**
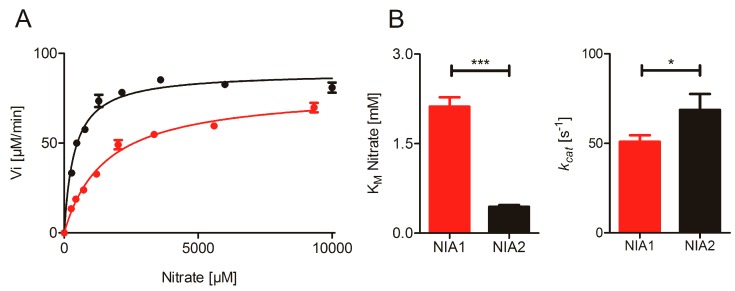
Nitrate reduction by NR-Mo-heme proteins. (**A**) Anaerobic Michaelis–Menten kinetics of NIA1-Mo-heme (red) and NIA2-Mo-heme (black) measured with the MV:nitrate assay. (**B**) Kinetic parameters of multiple batches of NIA1-Mo-heme (red) and NIA2-Mo-heme (black) determined in the MV:nitrate assay. The K_M_ and *k_cat_* for NIA1-Mo-heme and NIA2-Mo-heme were compared via unpaired t-test (GraphPad Prism 5). The means ± SEM of *n* = 33 kinetic series for NIA1-Mo-heme (made with 23 protein batches) and *n* = 13 kinetic series for NIA2-Mo-heme (eight protein batches used) are shown. *p*-value: *** < 0.001 < ** < 0.01 < * < 0.05.

Following successful purification of NIA1-Mo-heme and NIA2-Mo-heme, we first performed the nitrate reduction assay with reduced MV at different pH values and confirmed that NIA1 has the same pH-optimum at pH 7.0 as NIA2 and is also comparable to other NRs, e.g., from spinach [39,40] (Figure S1). Subsequently, we determined the steady-state kinetic parameters for a range of nitrate concentrations (Figure 1), yielding a K_M_^nitrate^ = 2120 ± 160 µM for NIA1-Mo-heme, which is approximately fivefold higher than the K_M_^nitrate^ for NIA2-Mo-heme (443 ± 26 µM), whereas the turnover number *k_cat_* for NIA1-Mo-heme (51 ± 4 s^−1^) is slightly but significantly lower than the one for NIA2-Mo-heme (69 ± 9 s^−1^). These results reveal distinct catalytic efficiencies *k_cat_*/K_M_ of 24 s^−1^ mM^−1^ for NIA1-Mo-heme and 155 s^−1^ mM^−1^ for NIA2-Mo-heme indicating that NIA2 is a far ‘better’ nitrate reductase, which can be mainly attributed to the lower K_M_ exhibited by NIA2.

### 2.2. Re-Constitution of Full-Length NR activity In Vitro

While full-length NIA2 can be obtained in high purity by recombinant expression in *Pichia pastoris* cells [41,42], the expression of full-length NIA1 in *Pichia pastoris* has been of limited success, yielding only trace amounts of protein (unpublished results). The expression strategy used here was, therefore, adjusted, and recombinant expression of both NIA1 and NIA2 protein was performed as two separate fragments in *Escherichia coli*—the N-terminal NR-Mo-heme fragment described above and a C-terminal fragment containing hinge 2 and the FAD-domain (residues 628–917 for NIA1-FAD, residues 626–917 for NIA2-FAD) (Figure S1).

**Figure 2:**
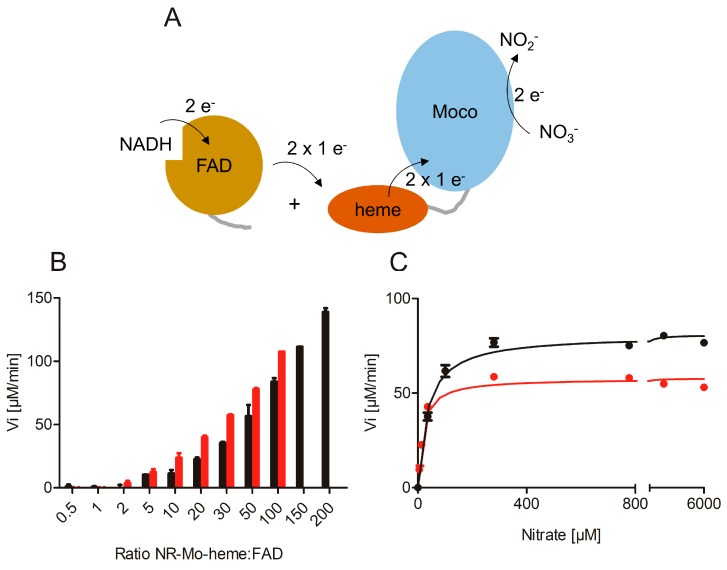
Re-constituted nitrate reductase activity. (**A**) Cartoon representation of the re-constitution of full-length NR activity by combination of the separate NR-Mo-heme and NR-FAD fragments in vitro. (**B**) Anaerobic NADH:nitrate assay of NR-Mo-heme (NIA1 red, NIA2 black) combined with increasing ratios of NR-FAD fragment. Increasing nitrate reductase activity was observed with increasing ratio of FAD-fragment. (**C**) Steady-state NADH:nitrate kinetics of re-constituted NIA1 (red) and NIA2 (black) activities.

Different ratios of NR-Mo-heme and NR-FAD (1:1–200) were mixed to restore the original electron transfer path using NADH and nitrate as substrates (Figure 2A). As the NR-FAD fragment was found to exhibit substantial diaphorase (NADH:O_2_ oxidoreductase) activity, the assay was performed under anaerobic conditions. With increasing NR-FAD concentrations, increasing enzyme-specific nitrate reductase activities were observed for both isoforms up to the maximal FAD concentrations of 10 or 20 µM (Figure 2B). Subsequently, we recorded nitrate-dependent NADH steady-state activity using an enzyme re-constituted with a ratio of 1:50 for both isoforms and found that the re-constitution of full-length activity was successfully achieved and that it was nitrate concentration dependent (Figure 2C). These kinetic series yielded an apparent *k_cat_* = 9.6 s^−1^ for composite NIA1, and *k_cat_* = 13.4 s^−1^ for composite NIA2. Both of these activities were somewhat lower compared to those obtained with MV as an electron donor and also compared to the reported parameters of full-length NIA2 (*k_cat_* = 33 s^−1^) [41]. This can be explained by the lack of covalent contact between the heme- and the FAD-domain, which required the use of an excess of FAD fragment to increase the interaction between the separated protein fragments. Consequently, the respective apparent K_M_ values were found to be lower due to the reduction in *k_cat_*, which is a result of the decreased electron transfer rate (apparent K_M_^nitrate^ = 17 µM for NIA1, 35 µM for NIA2).

### 2.3. Nitrite Reduction Activity

Reduced MV reacts non-enzymatically with nitrite at millimolar concentrations (Figure S2A). Therefore, an assay with an alternative electron donor had to be established for steady-state measurements of nitrite reductase activity. In contrast to MV, reduced benzyl viologen (BV) reacts non-enzymatically with nitrate, but is stable in the presence of nitrite at pH 7.5 (Figure S2), within the concentrations and time range required to conduct the experiments [43]. In initial tests, the optimal pH for nitrite reduction was determined for both NIA1- and NIA2-Mo-heme to be at pH 7.5 (Figure S3). Analogously to the MV:nitrate assay, the BV:nitrite assay had to be performed under anaerobic conditions to prevent non-enzymatic electron transfer from reduced BV to molecular oxygen. In contrast to MV which is known to donate electrons at the heme-domain [6], BV donates electrons directly to the Moco-domain. We could show this using a NIA2-Mo-heme mutant protein (H600A) with one of the heme-coordinating histidines mutated to an alanine, or a NIA1-fragment comprising only the Moco-domain, both of which showed nitrite reductase activity using BV as the artificial electron donor (Table 1).

**Figure 3:**
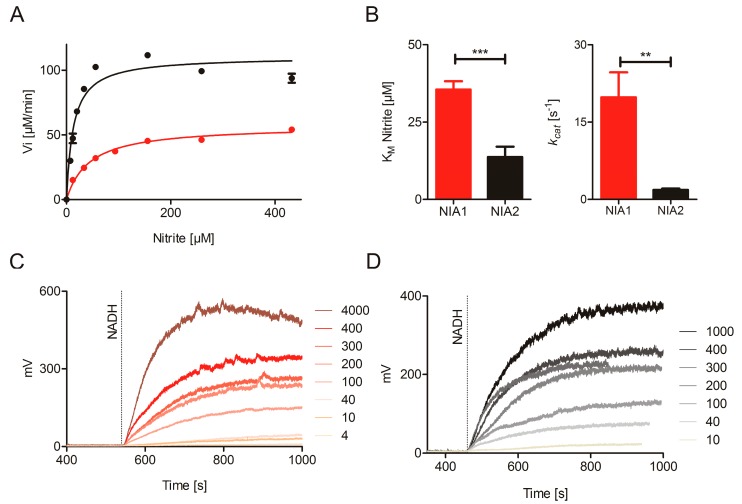

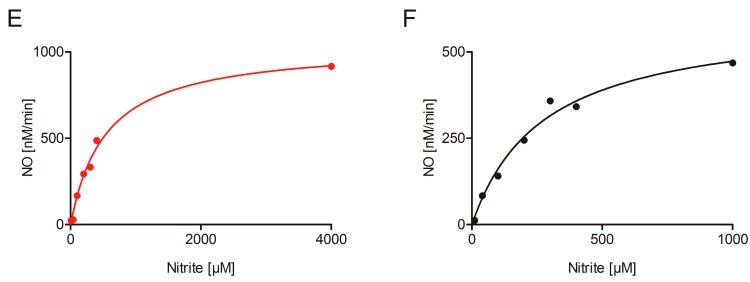
Nitrite reduction by NIA1 and NIA2. (**A**) Anaerobic Michaelis–Menten kinetics of 50 nM NIA1-Mo-heme (red) and 1 µM NIA2-Mo-heme (black) measured with the BV:nitrite assay. A higher concentration of NIA2-Mo-heme enzyme than NIA1-Mo-heme was needed to obtain reaction velocities in a similar order of magnitude. (**B**) Kinetic parameters of NIA1-Mo-heme (red) and NIA2-Mo-heme (black) determined in the BV:nitrite assay. The K_M_ and *k_cat_* for the NIA1-Mo-heme (red) and NIA2-Mo-heme (black) are compared via unpaired t-test (GraphPad Prism 5). The means ± SEM of *n* = 21 kinetic series for NIA1-Mo-heme (made with 12 protein batches) and *n* = 10 kinetic series for NIA2-Mo-heme (made with eight protein batches) are shown. *p*-value: *** < 0.001 < **< 0.01 < * < 0.05. (**C**,**D**) Nitrite reductase activity by re-constituted NIA1 (**C**) and NIA2 (**D**) measured using an NO-analyzer at different nitrite concentrations (indicated by the numbers, µM). (**E**,**F**) Hyperbolic curve fit of the assays from (**C**,**D**).

The kinetic parameters of the nitrite reducing activities with BV as electron donors are K_M_^nitrite^ of 35.5 ± 2.7 µM and 13.7 ± 3.3 µM and *k_cat_* values of 19.8 ± 4.8 s^−1^ and 1.8 ± 0.3 s^−1^ for NIA1-Mo-heme and NIA2-Mo-heme, respectively (Figure 3A,B, and Table 1) indicating that both proteins are able to act as nitrite reductases, with efficient substrate binding but slow turnover compared to the substrate nitrate. Nonetheless, their catalytic efficiencies differ significantly with 557 s^−1^mM^−1^ (NIA1-Mo-heme) and 131 s^−1^mM^−1^ (NIA2-Mo-heme). In this case, the difference in *k_cat_*, which is approximately ten-fold higher for NIA1-Mo-heme than for NIA2-Mo-heme, mainly accounts for NIA1 being the more efficient nitrite reductase.

To complement these results, an NO analyzer for direct quantification of the nitrite-dependent NO production by NR was used as it presents a very specific tool to record NO-release. However, despite its specificity for NO, this method can only give qualitative information about the kinetic parameters of enzyme-dependent NO production for two reasons: On the one hand, the NO is only quantified in the gas phase and not in the solution, where the reaction has taken place. This adds an unknown diffusion rate constant to the calculation. On the other hand, the weak interaction between the NR-Mo-heme and the NR-FAD fragments lowers the turnover number and consequently, also the K_M_. At least four different batches of NIA1-Mo-heme and NIA2-Mo-heme were re-constituted with 50-fold excess of the respective NR-FAD, and nitrite-concentration dependent NO production was measured in the presence of saturating NADH concentrations. All batches could efficiently produce NO down to very low nitrite concentrations (Figure 3C–E), thus, clearly confirming the enzyme-specific nitrite reduction by both composite NIA1 and NIA2. 

### 2.4. Nitrate Inhibition of Nitrite Reductase Activity

**Figure 4:**
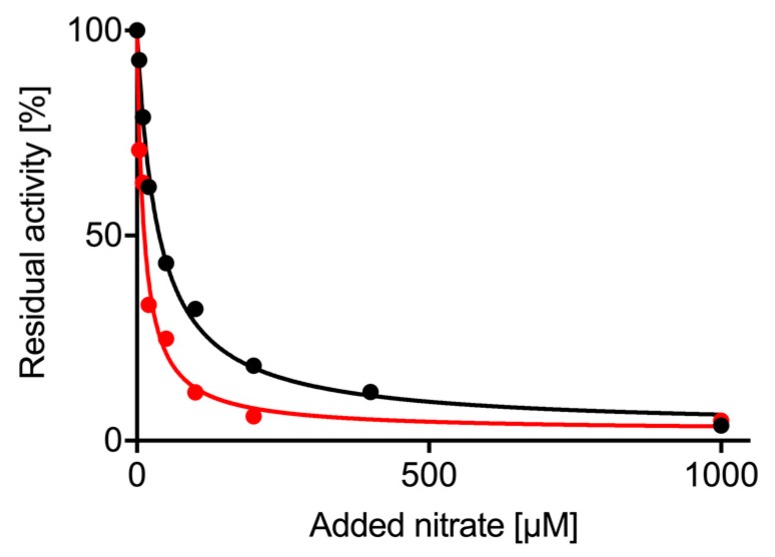
Inhibition of nitrite reductase activity by nitrate. Using re-constituted NR-activity, NO production by NIA1 (red) or NIA2 (black) was monitored on the NO analyzer with 400 µM nitrite. Increasing concentrations of nitrate were added simultaneously with the nitrite, and NO production decreased. The % residual activity was fitted with a hyperbolic curve (GraphPad Prism 7) and IC_50_ and I_max_ for nitrate determined.

The NO analyzer and the re-constituted NRs allowed us to measure the impact of nitrate on nitrite reduction as a competing substrate for NR. This experiment cannot be performed using one of the viologen assays due to their non-enzymatic reaction with either substrate. Using a saturating nitrite concentration of 400 µM, increasing concentrations of nitrate (0–1 mM) were added to the reaction mix and the amount of NO produced over time was measured (Figure 4). The nitrate concentration resulting in half-maximal inhibition of NO generation rates was IC_50_ = 12 ± 1.7 µM for NIA1-Mo-heme and 36 ± 2.7 µM for NIA2-Mo-heme with a maximal inhibition of up to 97% for both isoforms. This confirms that nitrate is a potent inhibitor for both isoforms which is able to efficiently impair nitrite reduction already at nitrate concentrations that are far below the respective K_M_^nitrate^ values for NIA1 and NIA2.

## 3. Discussion

Nitrate reductase was originally recognized as the enzyme catalyzing the eponymous reaction of nitrate reduction, the first step in plant nitrogen anabolism from the inorganic nutrient nitrate [44]. Decades of research have been dedicated to examining this very important and tightly regulated process in the plant. The discoveries that NR is involved in NO synthesis and also in NO scavenging, are by comparison new but probably no less important [8,28]. 

The role of NR in plants is further complicated in many plant species by the existence of two or even more isoforms of NR, as well as the observation that NR may undergo various post-translational modifications, such as phosphorylation [3,4] or sumoylation [45], which may affect its activity. Only in soybean, have the different isoforms (some constitutively expressed, some inducible ones) been comparatively analyzed with respect to their catalytic properties in nitrate and nitrite reduction, revealing that there are significant differences between the isoforms that may result in distinct functions *in planta* [46,47,48]. Other studies on NR activity did not differentiate between the isoforms, when analyzing NR purified from a plant species comprising more than one isoform (e.g., from corn [24] or tobacco [20]). In particular, the individual isoforms of *Arabidopsis thaliana*, NIA1 and NIA2, have to our knowledge not yet been separately purified or recombinantly expressed and compared to date. It has been reported, however, that the NR isoforms have individual expression patterns that are distinctly affected by environmental conditions [29,30,31]. Furthermore, it has been reported that the NR isoforms may have distinct roles in *Arabidopsis*, e.g., stomatal closure that is mainly mediated by NO from NIA1 [34] or that the majority of nitrate-reducing activity is performed by NIA2 [32], but similar contributions to NO formation by both isoforms have also been described [35].

Therefore, the aim of our study was to analyze the functional properties of the recombinantly expressed NIA1 from *Arabidopsis thaliana* in comparison to the properties of NIA2 to reveal whether the distinct functions in the plant are due to distinct isoform-specific kinetic properties or due to specific expression and activation state of either isoform in different plant tissues. Using our well-defined in vitro activity assays, we were able to measure the substrate-dependent velocities without any inhibiting effects/modulators that might be present when using (partially) purified enzyme from plant tissue.

The nitrate-reducing activity measurements using reduced MV as electron donor revealed a large and significant difference between the K_M_^nitrate^ for NIA1-Mo-heme and NIA2-Mo-heme. These values are in a similar range to the different K_M_ values for the soybean NR isoforms [46] and result together with the turnover numbers in a six-fold higher catalytic efficiency (*k_cat_*/K_M_) of *Arabidopsis* NIA2 for nitrate as compared to NIA1. This lower catalytic efficiency of NIA1 is consistent with the observation that *nia2* knockout plants retained only about 10% of nitrate reduction activity [49]. However, considering the physiological cytosolic nitrate concentrations that lie in the low millimolar range [50,51,52,53], NIA1 also has the ability to act as an efficient nitrate reductase, which is manifested in *nia2* single-knockout plants that grow with a normal phenotype [49]. Furthermore, we found that both isoforms clearly prefer NADH over NADPH as a substrate (Figure S4), which indicates that the catalytic efficiencies are mainly due to differences at the catalytic site at the Moco-domain, and not at the FAD-domain where NAD(P)H binds. 

To assess the nitrite-reducing capabilities of the *Arabidopsis* NR isoforms, we used two different methods. We first established an anaerobic assay using reduced BV as electron donor for the Moco domain of NR and nitrite as substrate. Measurement of the nitrite-reducing activity of NR using reduced BV is the first reported steady-state assay allowing direct and continuous measurement of the initial nitrite-dependent velocities of NO synthesis by NR and may also be useful for testing nitrite-reducing activity of NRs from other plant species in future. First, it has the advantage that reduced BV is stable in the presence of nitrite, in contrast to MV, which has been used previously to monitor nitrite reduction [28], but which reacts non-enzymatically with nitrite and, thus, causes significant background activity making it difficult to determine the nitrite-reduction velocities with varying nitrite concentrations. Second, the re-oxidation of BV due to electron transfer to the oxidized Mo center following nitrate reduction is directly monitored via a spectral change at 595 nm, which contrasts with the indirect NO quantification via the NO analyzer, in which the produced NO in the gas phase over the reaction mix is quantified and, thus, yields inexact NO synthesis rates. Nevertheless, as the NO analyzer specifically detects the released product NO, it serves as an important complementary method to confirm that the consumption of nitrite by NR indeed leads to NO formation.

The kinetic parameters of in vitro nitrite reducing activity determined with reduced BV clearly denote NIA1 as the more efficient nitrite reductase with a more than fourfold better catalytic efficiency compared to NIA2. The K_M_^nitrite^ values (35.5 µM for NIA1, 13.7 µM for NIA2) are considerably lower than those for nitrate, and are in a range similar to the physiological cytosolic nitrite concentrations. Nitrite concentrations in the plant cytosol may vary by two orders of magnitude depending on the environmental conditions but were determined not to exceed the low micromolar range [24,54]. In contrast to earlier reports, which described a significantly higher K_M_^nitrite^ for plant NR [24], the here determined values allow both NIA1 and NIA2 to bind nitrite as a substrate under physiological conditions.

Interestingly, very low nitrate concentrations are already sufficient to efficiently inhibit nitrite reductase activity of either NR isoform up to 97% (IC_50_^nitrate^ = 12 µM for NIA1, 36 µM for NIA2), which are roughly 200- and 10-fold lower than the K_M_^nitrate^ values for the respective isoforms. In light of the similarity of both substrates, a competitive inhibition mechanism by nitrite appears most likely.

Consequently, the question arises whether NR is at all able to directly produce NO under physiological conditions when nitrate is usually the much more highly centrated substrate compared to nitrite in the cytosol. The recent findings in *Chlamydomonas* [22] would support the hypothesis that NR may rather act in complex with NOFNiR as an indirect NO synthase, by donating electrons via its FAD-domain to NOFNiR which reduces nitrite to NO. However, this activity in higher land plants has not yet been confirmed. And if it were the case, it is as yet unclear what would trigger the switch in the electron transfer chain from the intra- to an inter-molecular pathway. In case of NIA2, it is possible that this trigger could be represented by a 14-3-3 protein binding to phosphorylated NIA2, which quickly inhibits the nitrate-reducing activity of NIA2 when the nutrient nitrate or reducing equivalents for the N assimilation become limiting. Then, binding of the 14-3-3 protein impairs the electron transfer from the heme cofactor to Moco by steric hindrance [5]. This would allow an immediate switch in the electron transfer pathway to NOFNiR, THB1 or other proteins yet to be identified, as the FAD-domain function is apparently not affected by the 14-3-3 protein binding. However, as no 14-3-3-mediated inhibition of NIA1 has been described until now, this trigger would be limited to NIA2, which would contrast with several reports that propose that NIA1 is the predominant isoform involved in NO synthesis [34,55,56]. More experiments are needed to support or refute this hypothesis: On the one hand, in vivo studies with mutant plants are needed that focus on the NR-NOFNiR interplay. On the other hand in vitro experiments with purified NIA1 are needed to analyze the impact of phosphorylation and 14-3-3 binding on NIA1 as well as with purified NIA1 and NIA2 to analyze the putative interaction with NOFNiR to produce NO.

Assuming that NR (NIA1 and/or NIA2) does not interact with NOFNiR, but is instead able to act as an NO synthase, leads again to the question how the two functions of NR are triggered *in planta*. Several arguments may help to answer this question: First, while nitrate reduction by NR is a crucial reaction for higher land plants, as growth and, thus, survival of the plant largely depends on the availability of nitrogen as a nutrient, the signaling molecule NO is only needed in trace amounts, so a rather slow NO synthesis rate by NR should be sufficient to meet the plant’s demands. Second, NO release is usually associated with a spike in nitrite concentration in the tissue, such as upon transition from light into darkness [20,24]. This would point to the fact that a local increase in nitrite concentration enhances the nitrite-reducing activity of NR as nitrite competes with nitrate for binding in the catalytic site. Third, tiny local changes in pH might also play a role in rendering nitrite the substrate for NR: In our in vitro system we determined the pH optimum for nitrate reduction to be at pH 7.0, whilst the pH optimum for nitrite reduction was at pH 7.5 (Figures S1C and S3) suggesting that a slight pH increase might push the function of NR from nitrate reductase towards nitrite reductase activity. These factors in combination with isoform-specific differences in expression, protein activation and their distinct kinetic properties described here might be the determinants for NR to act as a nitrate or nitrite reductase. 

Finally, with the successful re-constitution of nitrate reductase activity by mixing two NR fragments, we could demonstrate that the second linker of NR (hinge 2) is not essential for electron transfer from the FAD to the heme cofactor. This is consistent with our previous studies analyzing the electron transfer from FAD to heme in different viscous solutions that indicated no domain movement during intramolecular electron transfer [5] but is in contrast to a previous proposal that hinge 2 is essential for electron transfer activity within NR [57]. The interaction between the FAD and heme domains in the composite NR appears to be rather weak, resulting in activities below the maximum possible compared to full-length NR. Nonetheless, the successful restoration of both nitrate and nitrite reducing activities being able to use the physiological substrate NADH opens up new possibilities to study the structure, activity, and regulation of NR.

Taken together, this study presents the first comparison of the functional properties of the NR isoforms in *Arabidopsis thaliana* demonstrating that NIA2 functions mainly in nitrate reduction and NIA1 mainly in NO synthesis. However, more studies are needed to elucidate the complex interplay of nitrate reduction and nitrite reduction, in particular, whether an interaction with NOFNiR takes place, and the regulation of these processes in vitro as well as in vivo.

## 4. Materials and Methods 

### 4.1. Recombinant Proteins

The N-terminal fragment of *Arabidopsis thaliana* NIA2 (AGI code: AT1G37130) (NIA2-Mo-heme) was expressed in *E. coli* TP1004 (kindly provided by Tracy Palmer, Newcastle University, UK) using the plasmid described before [5] in LB-medium supplemented with ampicillin (100 µg/mL), kanamycin (25 µg/mL), sodium molybdate (1 mM), magnesium chloride (2 mM), and iron (III) chloride (10 µM) incubated at 37 °C to an OD_600_ of 0.2 to 0.4 and then induced by the addition of 50 µM isopropyl-ß-d-thiogalactoside (IPTG). The culture temperature was reduced to 18 °C and culture was continued for 70 h, then harvested by centrifugation. All subsequent steps were performed at 4 °C and all buffers for immobilized metal affinity chromatography (IMAC) were supplemented with COmplete^TM^ EDTA-free protease inhibitor cocktail (Roche, Mannheim, Germany). Cells were re-suspended in 10 mL lysis buffer (50 mM potassium phosphate pH 7.0, 200 mM sodium chloride) per gram wet cells and frozen at −80 °C. The suspension was then thawed and lysed using a Sonifier 250-D (BRANSON Ultrasonics Corporation, Danbury, CT, USA) and an EmulsiFlex-C5 (Avestin Europe GmbH, Mannheim, Germany). The raw lysate was supplemented with 10 µM hemin (from a 1 mM stock in 20 mM sodium hydroxide) [58]. Ni-NTA chromatography was performed in-batch for 30 min as the manufacturer describes (HisPur^TM^, Thermo Scientific, Rockford, IL, USA). After pouring the resin into a column, a wash with lysis buffer including 5 mM imidazole was performed to remove unspecifically bound proteins. For elution, the imidazole concentration was increased to 200 mM. The deep-red NIA2-Mo-heme-containing fractions were pooled and subjected to size exclusion chromatography (SEC) using an Äkta Prime system (GE Healthcare Europe GmbH, Freiburg, Germany) using a 16/60 Superdex 200 prep grade column (GE Healthcare Europe GmbH) and SEC buffer (20 mM Tris/hydrochloric acid pH 7.5, 200 mM sodium chloride, 10 mM magnesium acetate, 0.05% Tween 20). The protein peak eluting at about 60 mL was pooled. The concentration of heme-containing protein was determined via absorption at 413 nm using the extinction coefficient Ɛ_413_ = 120,000 M^−1^ cm^−1^. Molybdenum co-factor (Moco) saturation was quantified after oxidation to Form A and subsequent HPLC analysis by comparison to a Form A standard as described [59]. Protein was shock frozen in droplets in liquid nitrogen and stored at −80 °C.

Using the RAFL plasmid pda08083 (RIKEN BRC, Ibaraki, Japan) as a template for the *NIA1* gene (AGI code: AT1G77760), the sequence corresponding to *Arabidopsis thaliana* NIA1-Mo-heme fragment (residues 1–627) was PCR-cloned into the SphI and SalI restriction sites of pQE80L plasmid. The expression was similar to NIA2-Mo-heme with the following differences: Growth phase and expression of the transformed cells was at 25 °C. Induction was at OD_600_ = 0.4 with 100 µM IPTG for a duration of 20 h. The pH of the lysis, wash and elution buffers was adjusted to 7.5. Wash of the immobilized metal affinity chromatography (IMAC) column was performed after addition of 20 mM imidazole to the lysis buffer, while for the elution step, 250 mM imidazole was added. After SEC, the fractions containing non-degraded NIA1-Mo-heme were pooled, and after cofactor quantifications, the protein was shock-frozen in aliquots and stored at −80 °C.

The gene sequence corresponding to the NIA1-Mo fragment (residues 1–488) was PCR-amplified from the pQE80L-NIA1-Mo-heme plasmid and cloned into the KpnI and SalI restriction sites of pQE80L. Growth and expression were in *E. coli* TP1004 as described above for NIA2-Mo-heme, but with 20 µM IPTG for induction at 30 °C and 30 h. The cells were suspended (1 g/10 mL) in lysis buffer (50 mM potassium phosphate pH 7.5, 200 mM sodium chloride, 10 mM dithiothreitol, 1 mM sodium molybdate, 10 mM imidazole, COmplete^TM^ EDTA-free protease inhibitor cocktail). After one freeze-thaw cycle, the cells were lysed using an EmulsiFlex (Avestin). The His-tagged protein was first affinity-purified and then applied to an SEC as described for NIA2-Mo-heme. The NIA1-Mo peak was pooled, concentrated using an Amicon concentrator (Merck, Darmstadt, Germany) and Moco quantification via Form A, shock-frozen in aliquots and stored at 80 °C.

The NIA2-Mo-heme-H600A variant was expressed and purified as described elsewhere [5].

The DNA for the FAD-domains of NIA1 and NIA2 were PCR-amplified out of the respective full-length NR DNA sequences and had restriction sites introduced (BamHI and HindIII for NIA1-FAD, and PstI and HindIII for NIA2-FAD) for cloning into pQE80L plasmid. Expression was performed in *E. coli* BL21 Rosetta (Novagen, Darmstadt, Germany) using the same conditions for both FAD-fragments. Transformed cells were cultured at 37 °C to an OD_600_ of 0.4 and then induced by the addition of 400 µM IPTG. Induction was for 4 h at 37 °C. FAD-lysis buffer composition was 50 mM potassium phosphate pH 7.0, 200 mM sodium chloride, 5 mM imidazole, and COmplete^TM^ EDTA-free protease inhibitor cocktail. Wash of the IMAC was performed using the lysis buffer supplemented with 20 mM imidazole, elution buffer was with 250 mM imidazole. After elution, buffer exchange was performed using PD-10 columns (GE Healthcare) and SEC buffer. Concentration determination was based on the FAD-cofactor specific absorption at 450 nm and using an extinction coefficient Ɛ_450_ = 11,300 M^−1^ cm^−1.^

The DNA for the FAD-domains of NIA1 and NIA2 were PCR-amplified out of the respective full-length NR DNA sequences and had restriction sites introduced (BamHI and HindIII for NIA1-FAD, and PstI and HindIII for NIA2-FAD) for cloning into pQE80L plasmid. Expression was performed in *E. coli* BL21 Rosetta (Novagen, Darmstadt, Germany) using the same conditions for both FAD-fragments. Transformed cells were cultured at 37 °C to an OD_600_ of 0.4 and then induced by the addition of 400 µM IPTG. Induction was for 4 h at 37 °C. FAD-lysis buffer composition was 50 mM potassium phosphate pH 7.0, 200 mM sodium chloride, 5 mM imidazole, and COmplete^TM^ EDTA-free protease inhibitor cocktail. Wash of the IMAC was performed using the lysis buffer supplemented with 20 mM imidazole, elution buffer was with 250 mM imidazole. After elution, buffer exchange was performed using PD-10 columns (GE Healthcare) and SEC buffer. Concentration determination was based on the FAD-cofactor specific absorption at 450 nm and using an extinction coefficient Ɛ_450_ = 11,300 M^−1^ cm^−1.^

### 4.2. SDS-PAGE and Western Blot

Protein samples were separated by SDS-PAGE [60] and visualized by Coomassie Brilliant Blue G250 staining [61]. Proteins for Western blotting were transferred after PAGE to a PVDF membrane [62] using a semi-dry blotter, blocked with fat-free milk powder solution in TBST buffer (20 mM Tris, 150 mM sodium chloride, 0.1% Twee 20) and probed using polyclonal NR-specific antibody diluted 1:10,000 (AS08310, Agrisera, Vännäs, Sweden) and as secondary anti-rabbit horse radish peroxidase-coupled antibody (1:5000 dilution, Thermo Scientific). 

### 4.3. Enyzme Assays

All enzyme assays were performed in an anaerobic chamber at 22 °C to 25 °C (Coy Laboratory Products, Grass Lake, MI, USA), and enzyme-free negative controls were included in all experiments to confirm the enzyme-specific activities. For each single data point, three technical replicates were measured.

The MV:nitrate assay was performed with NIA1- or NIA2-Mo-heme as described [5] in a modified assay buffer (50 mM MOPS pH 7.0, 50 mM potassium chloride, 5 mM magnesium acetate, 1 mM calcium dichloride) in 96-well plates (Greiner-bio-one, Kremsmünster, Österreich) using a Sunrise plate reader (Tecan, Männedorf, Switzerland). Twenty-five nanomolar cofactor-saturated NR-Mo-heme protein was used in a final volume of 120 µL in the well. The slope of oxidizing MV was monitored at A_595_, and the initial velocities v_i_ were calculated, with 2 mole MV consumed for 1 mole nitrate. Triplicate values were used to determine mean and standard error of the mean (SEM) and then plotted and fitted in GraphPad Prism 5 using the Michaelis–Menten curve fit to yield *k_cat_* and K_M_ values. Activity assays were performed with multiple protein purification batches on multiple days (*n* = 33 for NIA1-Mo-heme and *n* = 13 for NIA2-Mo-heme).

The NADH:nitrate assay was performed using re-constituted NR. For re-constitution of NR activity, 100 nM NR-Mo-heme and 50 nM–10 or 20 µM NR-FAD (NIA1 and NIA2, respectively) were mixed in the pH 7.0 assay buffer (see above) in 96-well plates. Due to volume limitations in the experimental setup, we could not exceed a ratio of 1:100 for composite NIA1. The reaction was started by the addition of pre-mixed nitrate and NADH at a final saturating concentration of 220 µM (calculated based on its absorption at 340 nm and Ɛ_340_ = 6220 M^−1^ cm^−1^. For determination of the optimal Mo-heme:FAD ratio, a constant nitrate concentration of 2 mM was provided. For steady-state kinetic studies a range of nitrate concentrations from 0 to 6 mM were used and a constant FAD concentration of 5 µM (=50-fold excess). All measurements were performed in triplicate, and multiple NR-Mo-heme protein batches were used. The stoichiometric consumption of NADH was followed at A_340_, and initial slopes were determined using the Magellan software (Tecan) to calculate the v_i_ and further evaluated using GraphPad Prism. The comparison of the co-substrate NADH and NADPH were performed using 100 nM NIA1- or NIA2-Mo-heme supplemented with 5 µM of the respective NR-FAD fragment, 2 mM nitrate and 220 µM NADH or NADPH in assay buffer (pH 7.0) as described for the titration experiments.

The BV:nitrite assay was performed in a similar fashion to the MV:nitrate assay using the Mo-heme fragments. The pH optimum for nitrite reduction was shown to be pH 7.5 (Figure S3). Therefore, the buffer composition for nitrite reduction was: 50 mM MOPS pH 7.5, 50 mM potassium chloride, 5 mM magnesium acetate, 1 mM calcium dichloride. A nitrite dilution curve (0–435 µM) was prepared from anaerobic sodium nitrite powder fresh daily. Typically, 50 nM NIA1-Mo-heme and 500 nM NIA2-Mo-heme protein (unless otherwise indicated in the figure legends) were added to measure the initial slopes of stoichiometric re-oxidation of BV at A_595_. Activity assays were performed for multiple NR-Mo-heme purification batches on multiple days (*n* = 21 for NIA1-Mo-heme and *n* = 10 for NIA2-Mo-heme). The mean K_M_ and *k_cat_* ± SEM was determined using GraphPad Prism.

### 4.4. NO Quantification Using the NO-Analyzer

For nitric oxide quantification, an NO analyzer (Sievers 280i, Analytix, Boldon, UK) and modified assay buffer at pH 7.5 (as for BV:nitrite assay) was used, supplemented with Antifoam Y30 (Sigma, Saint Louis, MO, USA) at a dilution of 1:2000. An oxygen-free argon gas stream was bubbled through the glass reaction vessel containing the reaction components in a volume of 3 mL. The mixture was pipetted in the following order: First, buffer was placed in the vessel and the argon pressure adjusted to be equivalent to the vacuum coming from the analyzer. The vessel was closed and allowed to bubble and become anaerobic. After 4 min, anaerobic sodium nitrite solution to yield final concentrations of 10 µM to 4 mM (or nitrite + nitrate for inhibition experiments) was added from a sealed vial using a Hamilton syringe, followed at 6 min by anaerobic protein mix (100 pmol NR-Mo-heme + 5000 pmol NR-FAD). At 8 min, NADH solution was added to a final concentration of 220 µM to start the reaction. Steady-state NO release was recorded up to 20 min (or longer). 

For the evaluation, the areas under the steeply increasing start of the curve were determined (typically for 200 s) and converted to pmol NO by comparison with an NO standard curve that had been prepared as described elsewhere [63]. It was assumed that the amount of detected NO in the gas phase correlated with the concentration of NO in the solution. Therefore, by converting the amount of NO released from the 3 mL (at a given concentration of substrate nitrite) to NO concentration, resulted in an estimate of NO synthesis velocity (v_i_). By plotting this against the substrate concentration, a Michaelis–Menten-like plot was generated. For the determination of inhibition of nitrite reduction by nitrate, the NO synthesis velocity with 400 µM nitrite was set to 100% activity, and the reduced activities in the reaction samples were compared with this.

## Figures and Tables

**Table 1:** Kinetic parameters of nitrate reductase (NR)-fragments using the benzyl viologen (BV):nitrite assay.

Protein	K_M_^nitrite^ [µM]	*k_cat_*^nitrite^ [s^−1^]
NIA1-Mo-heme	35.5 ± 2.7	19.8 ± 4.8
NIA1-Mo	35.3 ± 3.2	137.5 ± 2.5
NIA2-Mo-heme	13.7 ± 3.3	1.8 ± 0.3
NIA2-Mo-heme-H600A	10.3 ± 1.2	1.2 ± 0.02

For the calculation of the means ± SEM, two kinetic series (1 protein batch) were used for NIA1-Mo; one kinetic series of one protein batch were used for NIA2-Mo-heme-H600A; see Figure 3 for NIA1-Mo-heme and NIA2-Mo-heme.

## Supplementary Materials

The following are available online at https://www.mdpi.com/2223-7747/8/3/67/s1, Figure S1: Recombinant proteins and pH optimum of nitrate-reducing activity with reduced MV as an electron donor. A. Purified NIA1-Mo-heme (0.8 µg) and NIA2-Mo-heme (2.2 µg) on 10% SDS-PAGE with Coomassie Brilliant Blue staining (C) and on a Western blot with anti-NR antibodies (WB). B. Purified FAD-fragment of NIA1 (3.6 µg) and NIA2 (0.9 µg) on 12% SDS-PAGE with Coomassie Brilliant Blue staining. C. The nitrate-reducing activity using reduced MV by NIA1-Mo-heme was monitored and had a pH optimum at pH 7.0. Figure S2: Unspecific re-oxidation of artificial electron donors (MV shown in blue, BV in brown) monitored at A595. A, B. Enzyme-free reaction mix of reduced MV, assay buffer and 0.157–200 mM nitrite (A) or nitrate (B). C, D. Enzyme-free reaction mix of reduced BV, assay buffer and 0.157–200 mM nitrite (C) or nitrate (D). Figure S3: Determination of pH optimum for the BV:nitrite steady-state kinetic assay. The pH optimum for both NIA1-Mo-heme (red, left panel) and NIA2-Mo-heme (black, right panel) for nitrite-reducing activity using reduced BV is at pH 7.5. Figure S4: Comparison of NADH or NADPH as substrates for composite NIA1 and NIA2. Initial velocities of 100 nM NIA1- (red) or NIA2- (black) Mo-heme supplemented with 5 µM of the respective NR-FAD at saturating nitrate and NADH or NADPH concentrations were recorded.
