# Supplementary figures and images for: Isoform-Specific NO Synthesis by Arabidopsis thaliana Nitrate Reductase
Source: Plants (Basel). 2019 Mar 16;8(3):67. doi: 10.3390/plants8030067 (PMC6473903; doi:10.3390/plants8030067)

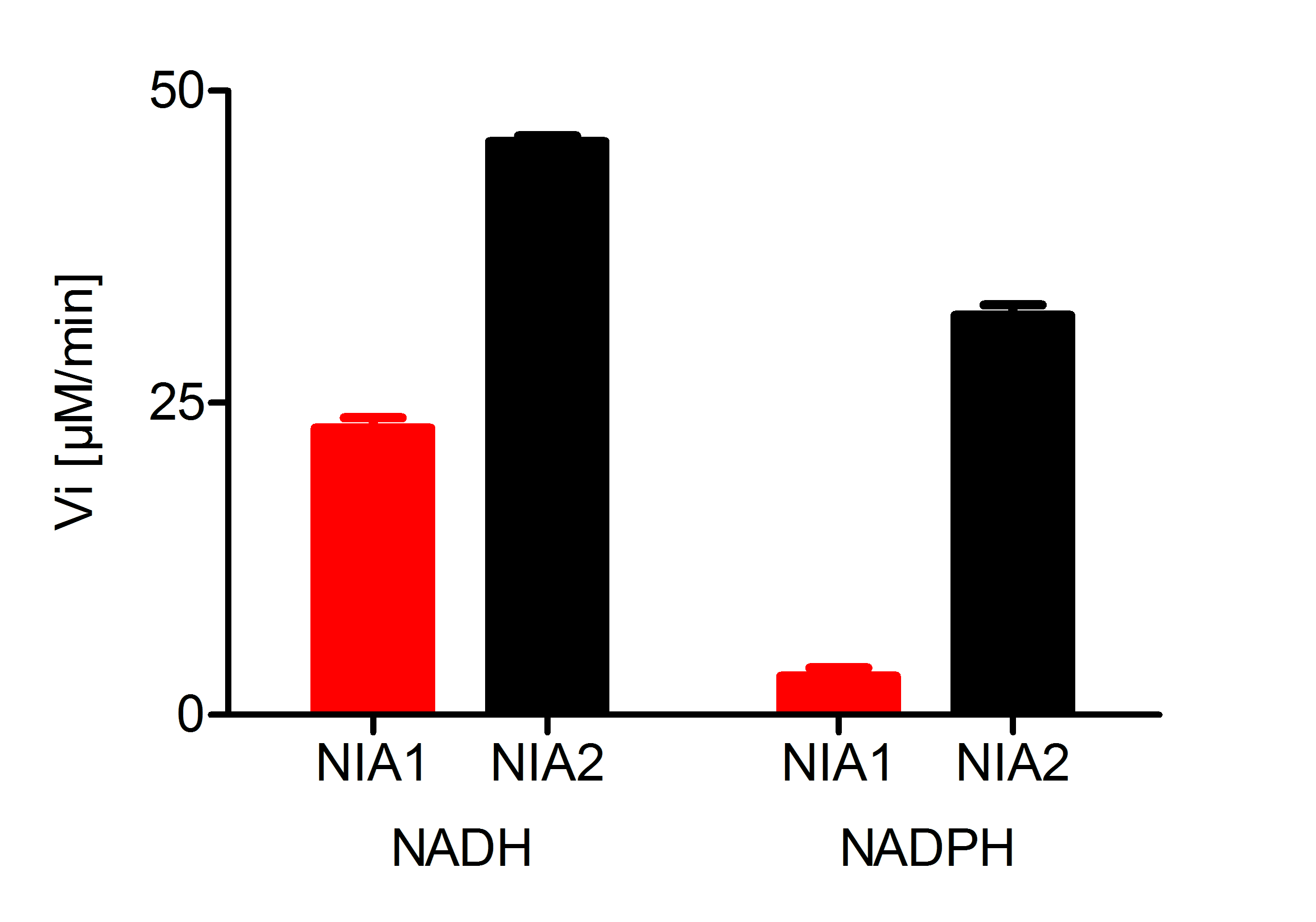

Supplement: Supplementary file 1 [file plants-08-00067-s001.zip › Figure S4.tif]

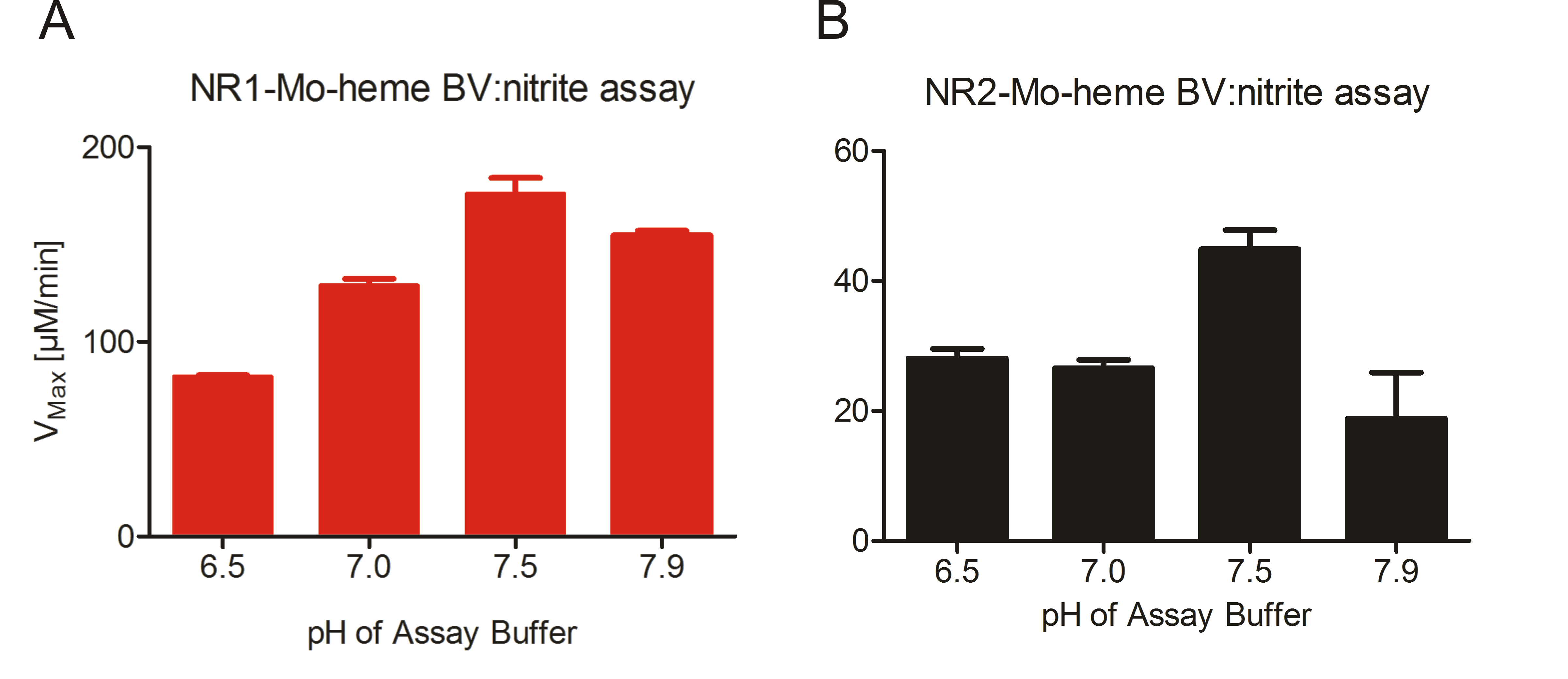

Supplement: Supplementary file 1 [file plants-08-00067-s001.zip › Figure S3.tif]

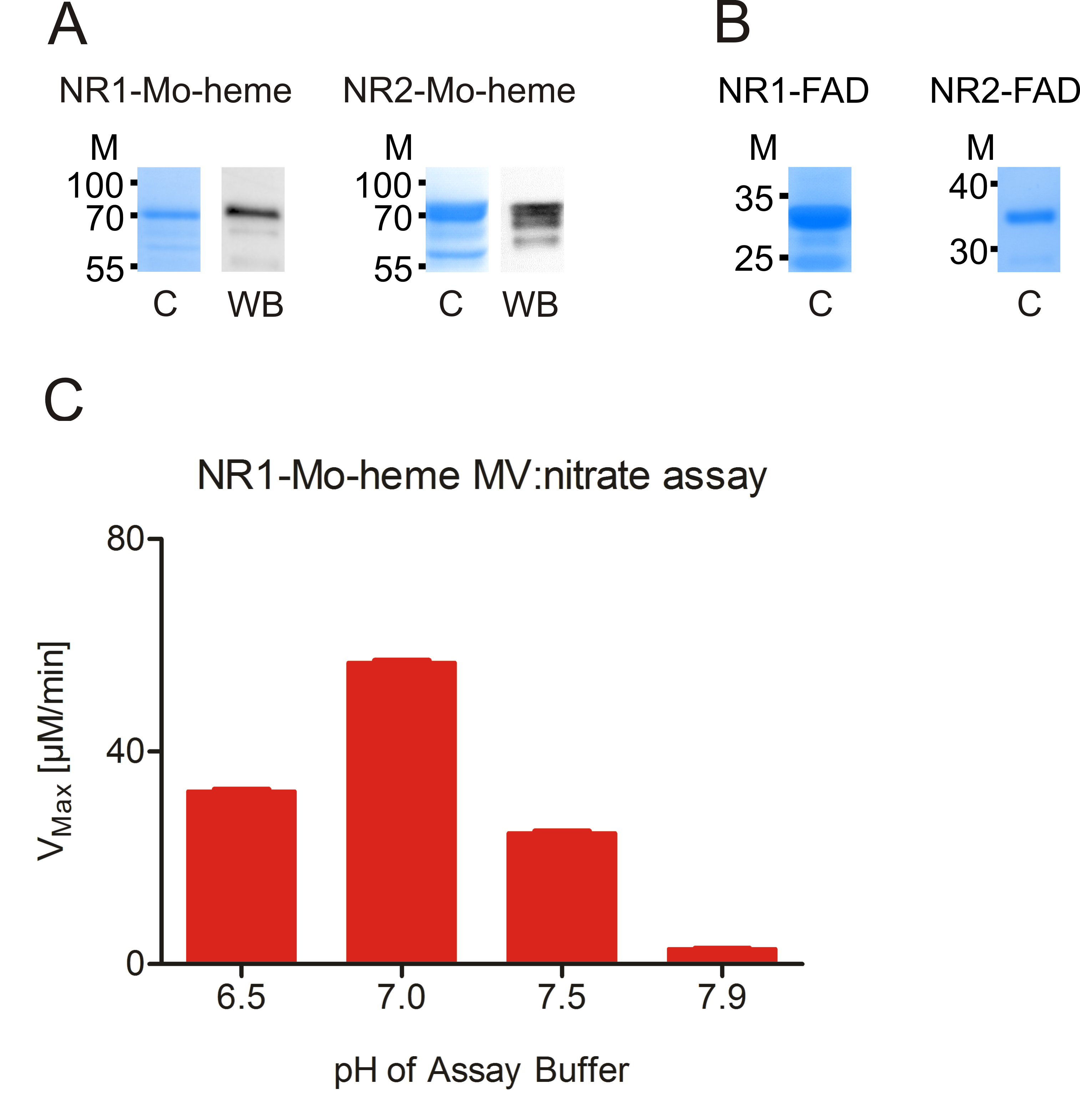

Supplement: Supplementary file 1 [file plants-08-00067-s001.zip › Figure S1.tif]
